# A Review: Using Ionic Liquids for Lignin Extraction from Lignocellulose and High-Value Utilization

**DOI:** 10.3390/molecules30122514

**Published:** 2025-06-09

**Authors:** Xinyu Li, Jiming Yang, Wei He, Shuangfei Zhao, Zheng Fang, Kai Guo, Yuguang Li

**Affiliations:** 1 College of Biotechnology and Pharmaceutical Engineering, Nanjing Tech University, Nanjing 211816, China; lixinyu@njtech.edu.cn (X.L.);; 2 State Key Laboratory of Materials-Oriented Chemical Engineering, Nanjing Tech University, Nanjing 211816, China; 3 Institute of Nanjing Advanced Biomaterials & Processing Equipment, Nanjing 211299, China

**Keywords:** lignin, lignin extraction, ionic liquids, lignin high-value utilization, lignocelluose

## Abstract

Lignocellulose is the most abundant renewable resource in nature, providing a large supply of lignin. The efficient separation and utilization of lignin from lignocellulose can help alleviate the current shortage of fossil fuels. Ionic liquids, as green solvents, have been widely applied in the field of biorefining. However, most research has focused on the extraction and purification of cellulose, while lignin is often treated as a by-product. The high-value utilization of lignin has currently emerged as a hot topic. This review summarizes recent advances in the extraction of lignin from lignocellulose using ionic liquids and the mechanisms of lignin extraction. Additionally, it briefly discusses the applications of ionic liquids in the high-value utilization of lignin, including lignin depolymerization, modification, the preparation of lignin-based functional materials, and biofuels. This review aims to provide ideas for the extraction and high-value utilization of lignin through ionic liquids.

## 1. Introduction

It is reported that approximately 815 billion tons of lignocellulose biomass are produced globally each year, yet only 4.5% is effectively utilized [1]. As the most abundant renewable resource, lignocellulose biomass has the potential to meet sustainable chemical and energy demands and reduce heavy reliance on fossil fuels [1]. Lignocellulose biomass has different resources and extremely complex structures. Primarily, it is composed of 35–50% cellulose, 20–35% hemicellulose, and 10–25% lignin, along with small amounts of proteins, lipids, pectin, and other substances [2]. Traditionally, lignin is regarded as waste in traditional biorefining processes and cannot be effectively utilized, with most research focusing on the purification and utilization of cellulose. However, lignin is the second most abundant biopolymer on Earth after cellulose and represents the most important renewable source of aromatic compounds to date [3]. In recent years, lignin has been increasingly recognized as a source of many industrially important materials. It can be converted into biofuels (such as syngas, phenolic oil, etc.), numerous value-added materials (carbon materials, hydrogels, nanocomposites, etc.), important industrial chemicals (such as benzene, vanillin, guaiacol, etc.), and macromolecules (including epoxy resins, dispersants, surfactants, etc.) [4,5,6]. More attention has been devoted to the high-value utilization of lignin, and the methods for lignin fractionation from lignocellulose have become increasingly diverse, such as autohydrolysis, steam explosion, and ionic liquids. With the continuous development of lignocellulose fractionation technology, researchers have proposed a new separation approach—the lignin priority method, which emphasizes maintaining the structural integrity of lignin during the separation process while achieving efficient utilization of lignin and carbohydrates [7]. This method has received widespread attention in the current field of biorefining. The structural tunability, high solubility, and catalytic activity of ionic liquids make them more capable of addressing the challenges of lignin extraction and lignin high-value utilization [4]. In this review, we discuss the research progress and existing challenges in the extraction and utilization of lignin from lignocellulose biomass using ionic liquids, putting forward the ideas for further optimizing the efficiency of lignin extraction and high-value utilization with ionic liquids.

## 2. Structure of Lignin

**Figure 1:**
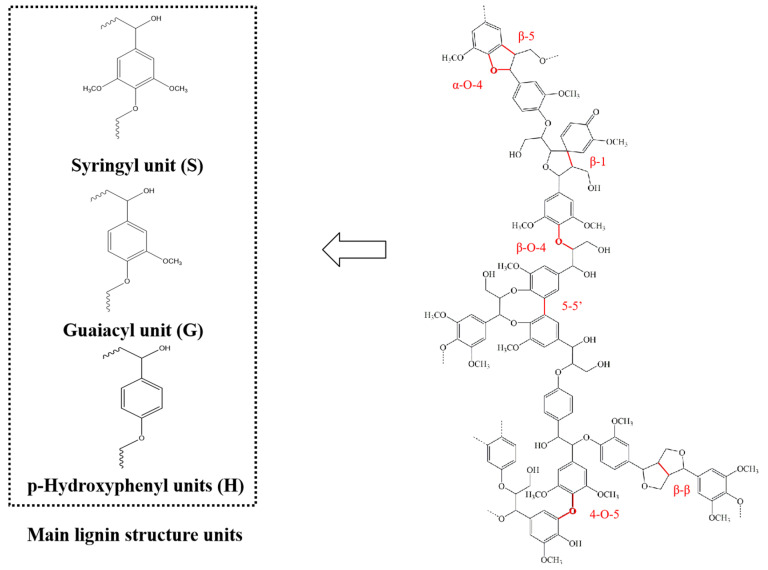
Lignin structure and main unit bonds [13].

Lignin is an amorphous aromatic polymer that is polymerized from three aromatic alcohols: p-coumarol, coniferol, and sinapic alcohol [8]. When polymerized as lignin, the units derived from monolignols are called p-hydroxyphenyl (H), guaiacol (G), and springyl (S) units, respectively [9]. Lignin has different relative proportions of lignin units in different plant species. Carbon–oxygen (ether) bonds and carbon–carbon bonds are the two main bonds connecting these units. The connection of the C-C bond, including β-β, β-5, and β-1 bonds, is very stable [10]. And β-O-4 is one of the main C-O bonds in lignin [11]. Figure 1 shows the main units and unit bonds of lignin. The presence of various C-C and C-O bonds hinders the degradation of lignin. Among them, the β-O-4 bond is the most abundant bond type between lignin units, so the breaking of the β-O-4 bond is the key step of lignin degradation [12].

## 3. Extraction of Lignin from Lignocellulose by Ionic Liquids

### 3.1. Technology of Lignin Extraction

The extraction technology of lignin from lignocellulose reported in the current study can be roughly divided into four categories: physical methods [14,15,16,17], chemical methods [18,19,20,21], physicochemical methods [22,23,24,25], and biological methods [26,27]. In Table 1, we listed some typical methods for lignocellulose pretreatment and compared the characteristics of methods [28,29]. Ionic liquids present a greener alternative for lignocellulose pretreatment due to their advantageous properties, such as weak corrosiveness, low vapor pressure, low melting points, and non-volatility [29]. Furthermore, the structural tunability of ionic liquids provides greater flexibility for optimizing their application in lignin extraction from lignocellulose.

Ionic Liquids (ILs) are salts composed of organic cations and inorganic or organic anions that remain liquid at or near room temperature. Common cations include imidazolium, pyridinium, quaternary ammonium, sulfonium, and phosphonium types, while typical anions comprise Cl^−^, Br^−^, AlCl_4_^−^, BF_4_^−^, NTf_2_^−^, CH_3_COO^−^, Gly^−^, etc. The intricate interplay of hydrogen bonding, coulombic interactions, and van der Waals forces between cations and anions confers ILs unique properties such as non-flammability, low vapor pressure, and high thermal stability [4,30]. As early as 2002, Richard P. Swatloski et al. [31] first reported the dissolution of cellulose using ionic liquids, marking the inception of ILs applications in lignocellulose pretreatment. Initially, ILs pretreatment primarily targeted cellulose extraction to enhance enzymatic saccharification efficiency. With advancing research, lignin extraction via ILs gradually gained attention. Pu et al. [32] demonstrated that [Bmim][MeSO_4_] most effectively dissolved lignin, while ILs with large, non-coordinating anions like [BF_4_]^−^ and [PF_6_]^−^ were unsuitable. Daan Glas et al. [33] compared lignin solubility in non-imidazolium ILs, revealing that N-butyl-N-methylpyrrolidinium dicyanamide ([BMPyr][N(CN)_2_]) exhibited a superior dissolution effect. It can be attributed to hydrogen bonding between the [N(CN)_2_]^−^ and lignin hydroxyl groups. Extensive studies have validated the lignin-dissolving capacity of ILs, laying the foundation for their application in lignocellulosic lignin extraction.

### 3.2. Process of Lignin Extraction

**Figure 2:**
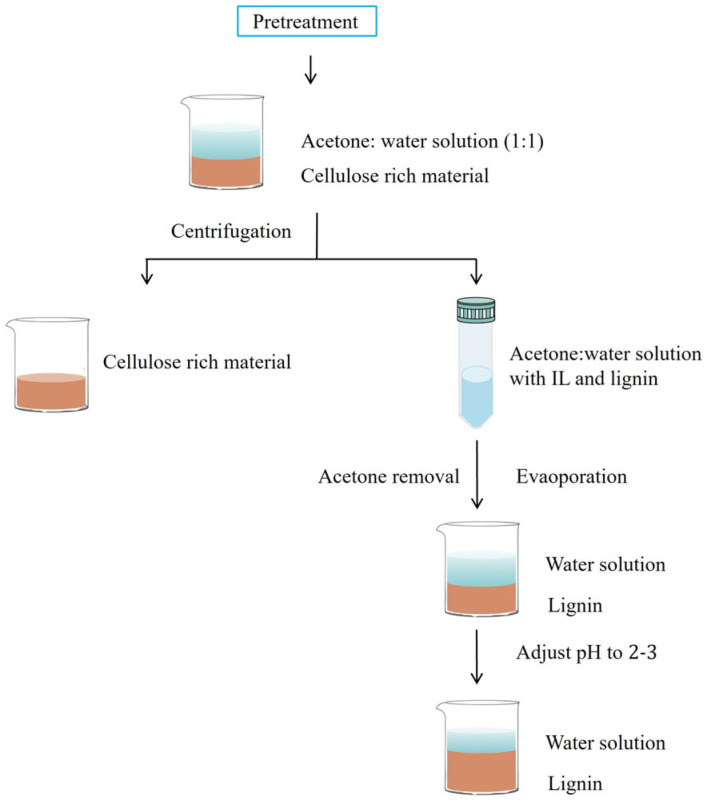
Lignin extraction process.

The extraction of lignin from lignocellulose typically exploits the differences in solubility among cellulose, lignin, and ILs in solvents, employing an anti-solvent fractional separation approach. Based on lignin’s good solubility in acetone–water (1:1 *v*/*v*) solutions, Sun et al. [34] proposed a widely adopted lignin recovery method (Figure 2): after pretreatment, an acetone–water mixture is first added to precipitate cellulose from the IL-biomass solution, followed by acetone evaporation to precipitate the lignin-rich fraction. Finally, acid can be added to adjust the solution’s pH to 2–3, protonating the phenolic hydroxyl and carboxyl groups of lignin to reduce the electrostatic interactions in the aqueous solution, thereby achieving precipitation of lignin macromolecules [35]. Most subsequent studies have followed this separation technique, with some variations in the choice of anti-solvent types [36,37]. However, during IL pretreatment, lignin often undergoes partial degradation into water-soluble fragments, and these low molecular weight lignin fragments may remain in the aqueous solution and cannot be completely precipitated. Additionally, the one-step anti-solvent precipitation method makes it difficult to effectively fractionate lignin by molecular weight. Currently, lignin separation is often conducted as an additional step in cellulose purification. Future work should further optimize lignin separation pathways to achieve efficient utilization of the lignin component in lignocellulose.

### 3.3. Aprotic Ionic Liquids (AILs)

Ionic liquids commonly used for lignocellulose fractionation can be divided into aprotic ionic liquids (AILs) and protic ionic liquids (PILs). In this section, we will summarize some ILs system for lignin extraction.

(1) Common AILs for lignin extraction

**Figure 3:**
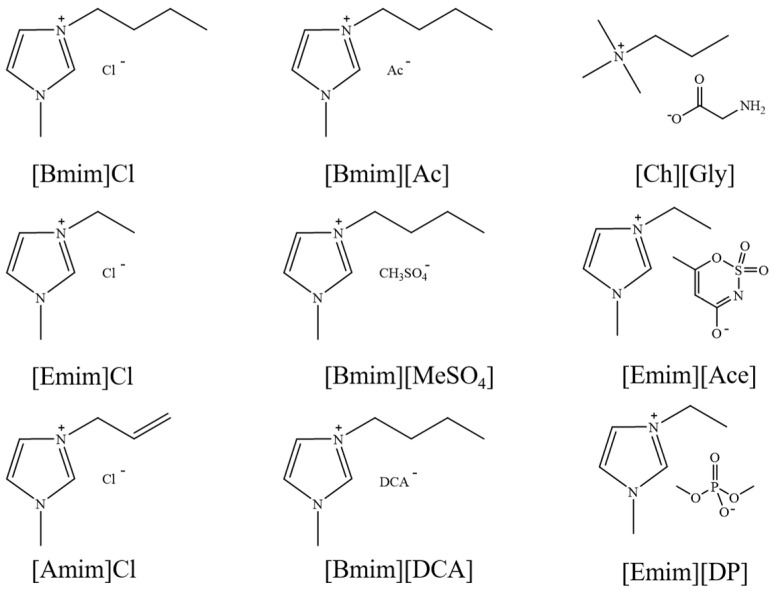
Commonly used AILs for lignin extraction.

Since 2002, aprotic ionic liquids have been extensively studied for their applications in lignocellulose fractionation. Strehmel et al. [38] compared imidazolium-based ionic liquids with chloride, acetate, methanesulfonate, and tosylate anions for lignin extraction from birch bark. Their results demonstrated that 1-butyl-3-methylimidazolium acetate ([Bmim][Ac]) exhibited the highest efficiency among the investigated ionic liquids for lignin extraction from bark. This high efficiency can be attributed to both the strong catalytic function of this ionic liquid in bond cleavage and the positive contribution of acetate ions to transesterification reactions. Ionic liquids such as [Bmim]Cl, [Emim][Ac], and [Bmim][DCA] have been reported for lignin extraction [38,39]. However, these imidazolium-based ionic liquids typically require extended pretreatment times and elevated reaction temperatures while yielding unsatisfactory lignin recovery rates. Subsequently, pyrrolidinium and pyridinium-based ionic liquids have been proposed to be used for more efficient lignin extraction [40,41]. Figure 3 show the common AILs used for lignin extraction.

**Figure 4:**
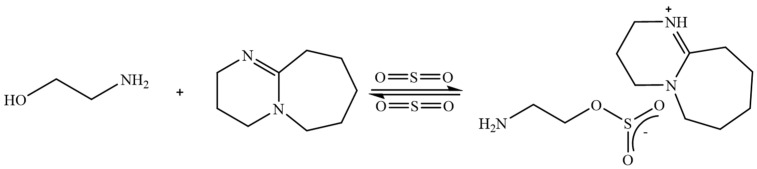
Schematic representation of DBU–MEA–SO_2_ SIL system [45].

Switchable ionic liquids (SILs), a class of ionic liquids with interesting reversible ability, are synthesized from molecular moieties, including organic superbases, lower and higher alcohols, and acid gases such as CO_2_ and SO_2_. These compounds can be reversibly converted back to their molecular precursors through processes like heating [41]. Swithable ionic liquids (such as DBU-MEA-SO_2_, DBU-glycerol-CO_2_, etc.) have been reported for selective lignin extraction from wood and industrial lignin materials [42,43,44,45]. Figure 4 shows the structure of one of the reversible ionic liquids used for lignin extraction. Compared to commercial ionic liquids, switchable ionic liquids are prepared from low-cost chemicals, resulting in significantly lower production costs. Furthermore, SILs can fractionate lignocellulosic biomass under milder conditions (120 °C) while preserving lignin’s native structure.

The lignin-first approach is a hotspot in current lignocellulose fractionation research [46,47]. This strategy aims to minimize structural modifications and condensation of lignin during the separation process while simultaneously enabling effective utilization of both cellulose and lignin. There are also few reports on the application of lignin priority strategy in the extraction of lignin by AILs [1,46,48]. Yang et al. [46] adopted lignin-first strategy and extracted lignin using NaOH and [C_2_C_1_Im][OAc] at 60 °C, resulting in lignin with higher average molecular weight and S/G value. Xu et al. [48] proposed a lignin-first separation approach using 1-ethyl-3-methylimidazolium acesulfamate ([Emim][Ace]) for lignocellulose, followed by a mild alkaline extraction process. This method achieved a 70.7% lignin extraction yield, with the extracted lignin exhibiting high purity and moderate molecular weight, making it suitable for high-value utilization.

It is worth mentioning that imidazole-based ionic liquids are not biocompatible and show toxicity to hydrolases and microorganisms, which limits their application to a certain extent. In recent years, biologically derived choline ILs have been reported to be developed, have been shown to selectively remove lignin, and are widely favored due to their biocompatibility with enzymes and even microorganisms [49,50,51,52,53,54]. Juliene Da Câ Mara Rocha et al. [53] reported the application of choline chloride ([Ch]Cl) in the pretreatment of green coconut fiber, which could extract 71.21% lignin. Wang et al. [54] prepared and synthesized five kinds of Ch-OCA-ILs, choline lactic ([Ch][LA]) presented the best lignin removal effect and could produce regenerated lignin rich in H units. Xu et al. [55] reported a cellulase-compatible imidazole-based ionic liquid (1-ethyl-3-methyl-imidazolium dimethylphosphate, EmimDP). In situ saccharification can be achieved by adding a certain amount of water after pretreatment, which reduces the washing process of lignocellulose after pretreatment and reduces energy consumption. However, due to its biocompatibility, ionic liquids may also be biodegraded during enzymatic hydrolysis, resulting in the consumption of ionic liquids.

(2) AILs-solvents system

Although ionic liquids have been widely used in lignin extraction, the higher viscosity of ionic liquids compared to organic solvents increases mass transfer resistance, making it difficult to achieve effective processing under high biomass solid loading conditions. This high-viscosity characteristic prolongs the time required to reach thermodynamic equilibrium (typically 12–24 h), thereby limiting pretreatment efficiency. To address this issue, researchers have proposed adding co-solvents to ionic liquids to reduce system viscosity while simultaneously decreasing ionic liquid usage and reducing costs. In recent years, numerous studies have focused on exploring the effectiveness of polar solvents in lignocellulosic pretreatment processes. Research results demonstrate that these solvents can disrupt the interchain structure of lignocellulose and enhance pretreatment efficiency [49,56]. Moreover, the combination of these polar solvents with ionic liquids reduces the viscosity of the pretreatment system, significantly improving mass transfer performance and increasing the solid loading of the reaction system.

At present, extensive research has focused on the pretreatment effects of the IL–water mixing system on lignocellulose materials [57,58]. Takashi Akiba et al. [59] found that adding small amounts of water could enhance lignin solubility in many polar ionic liquids. Although water addition reduced the proton acceptance capacity (β) of these ILs, their proton donation ability (α) was observed to increase, ultimately improving the ILs’ lignin dissolution capability. For ILs with β > 0.4, appropriate water addition was shown to increase lignin solubility in ionic liquids. However, Brandt et al. [60] observed that the pretreatment performance of ILs containing [MeSO_4_]^−^ anions deteriorated with water addition. There are contradictions in the literature about the role of water in the pretreatment efficiency of IL water solvent. Belesov et al. [61] demonstrated that adding DMSO (20%) to ILs significantly influenced lignin properties obtained by [Bmim][MeSO_4_], with DMSO preventing condensation processes and chemical modifications of biopolymers. Meanwhile, Jin et al. [62] introduced γ-valerolactone into ILs to form organic electrolyte systems (OES). The use of IL-based OES not only maintained pretreatment efficiency while reducing IL consumption but also mitigated the harsh pretreatment conditions required for pure IL systems, enabling an efficient selective delignification rate.

(3) AILs recycle

In addition, the high viscosity and high boiling point of ionic liquids hinder their recycling. The recovery process of ionic liquid system must rely on the addition of an antisolvent (such as water, ethanol, etc.) to realize the regeneration and recycling of ionic liquid. This process increases the complexity of operation but also increases the energy consumption and cost of recovery steps. These factors together limit the application prospect of ionic liquid pretreatment technology on an industrial scale. Manita Kuntapa et al. [63] developed a method of 1-ethyl-3-methylimidazole acetate-dimethyl sulfoxide ([Emim][OAc]-DMSO) pretreatment of corn straw, which improved the number of [Emim][OAc] recycling (up to 17 rounds) and enhanced the lignin removal rate. Yang et al. [64] proposed that the ionic liquid could be recycled by using 1-ethyl-3-methylimidazole tetrafluoroborate-arginine ([Emim][BF_4_]-Arg) system to carry out a simple filtration step for the reaction system. The total circulation times of the ionic liquid system could reach 5 rounds, reducing the recovery cost of adding water after a single pretreatment.

(4) AILs binary composite system

To improve the efficiency of ionic liquid pretreatment, researchers have also constructed a multi-element pretreatment system to improve the lignin extraction efficiency through the synergistic effect of multiple elements, which can overcome the shortcomings of a single solvent system [65,66,67,68]. Wei et al. [65] pretreated herbal residue biomass with a 1-butyl-3-methylimidazolium chloride/*p*-toluenesulfonic acid ([Bmim]Cl/TsOH) solvent system. The addition of low-concentration TsOH as an additive in the IL system can synergistically enhance the pretreatment performance. The system can not only avoid the corrosion of equipment during the use of high concentration of TsOH and also improve the efficiency of ionic liquid pretreatment. The yield of regenerated lignin can reach 68.3% at 140 °C. Based on 1-butyl-3-methylimidazolium chloride/hydrochloric acid ([C_4_C_1_im]Cl/HCl), Zhang et al. [66] studied the role of formaldehyde (FA) in the anti-condensation of lignin during the pretreatment of corn straw. Due to the aldehyde reaction between FA and lignin, the condensation of lignin fragments is inhibited, and high molecular weight highly soluble lignin can be obtained.

(5) Summary of this section

Currently, various AILs have been researched for lignin extraction. In addition to common imidazole-based ionic liquids, reversible ionic liquids, choline-based ionic liquids, etc., have been researched to meet the needs of low biological toxicity and cost reduction based on lignin extraction. Due to the wide variety of anions and cations in aprotic ionic liquids, the lignin extraction mechanism is complex and will be described in detail in Section 5. It did not elaborate in this section. With the continuous development of biorefining technology, higher requirements have been put forward for the utilization of lignocellulose, and lignin priority methods have become a hot topic in current research. The lignin priority strategy not only emphasizes the integrity of lignin structure, but also ensures the utilization efficiency of cellulose, achieving higher value utilization of lignocellulose. So far, there are some studies that have confirmed the feasibility of AIL in lignin priority strategies, and further research should be explored for the use of ionic liquids in lignin priority strategies. In addition to simply using ionic liquids, researchers have also constructed multi-component composite systems that can improve the efficiency of ionic liquid extraction. For example, adding solvents (DMSO, water) can compensate for the problems of high mass transfer resistance and energy consumption cost in the pretreatment process of non-ionic liquids. The synergistic effect of ionic liquids and other chemicals (TsOH) can improve the extraction efficiency of lignin in ionic liquid systems.

### 3.4. Protic Ionic Liquids (PILs)

Biomass pretreatment using IL initially focused on dissolving cellulose (aprotic) solvents, including the most commonly used 1-ethyl-3-methylimidazole acetate ([Emim][OAc]). Although aprotic ILs have high solubilization properties of lignin and hemicellulose, their practical application is limited by the disadvantages of high production cost, poor thermal stability, difficult recovery, and low humidity tolerance. In contrast, PILs formed by Bronsted base and acid neutralization reaction has a simpler synthesis process, lower production cost and faster production speed [69,70]. The PILs has a lower boiling point and can be recovered by distillation [71,72]. At the same time, due to its dissociatable protons, which can act as a donor of proton acids and hydrogen bonds, PILs destroys the bond between lignin and hemicellulose, and depolymerizes lignin by breaking β-O-4′ ether bonds, glycosidic bonds, and ester bonds, with high delignification capacity [73]. In addition, the pretreatment of lignocellulosic biomass using PILs offers the advantage of simultaneous lignin extraction and activation (increase in carbonyl groups), enabling its use in the synthesis of hybrid materials [74]. The above characteristics make PILs have great potential in biomass fractionation.

Amino, imidazolyl, and pyrrolyl proton ionic liquids have been developed for lignin extraction (Table 2). Most of the proton ionic liquids have no solubility to cellulose and can selectively separate lignin and polysaccharides.

Rabia Muazzam et al. [75] reported the application of a superbase ionic liquid with low biological toxicity in lignin extraction. The lignin removal rate of 81% could be achieved after pretreatment at 140 °C for 4 h. Through the characterization of regenerated lignin, Achinivu et al. [80] found that pyrrolidine acetate ([Pyrr][Ac]), which is more acidic, would promote the chain scission of the bonds between lignin units, resulting in lignin with smaller average molecular weight and more uniform dispersion. This indicates that the higher acidity in PILs will improve the lignin removal rate.

PILs pretreatment reduces the molecular weight of lignin through proton induced β-O-4′ bond cleavage, destroys the hydrogen bond network of lignin, enhances the solubility of lignin in ionic liquid, and realizes lignin separation. But the acidic environment will also lead to C-C condensation, which may increase the difficulty of lignin value-added utilization [73]. Chen et al. [84] proposed combining proton ionic liquid pretreatment technology with organic solvent pretreatment, and the added organic alcohol can induce α-alkoxylation, generate α-alkoxyether units, and prevent lignin from further condensation to form new C-C bonds. At the same time, the α-alkoxy ether unit increased the solubility of lignin in the solvent system and improved the lignin removal efficiency. In addition, the addition of organic solvent reduces the viscosity of the fractionation system and improves the processing capacity of the fractionation system (50%). And compared with water, organic solvents are more conducive to the recovery of IL system after fractionation and reduce the recovery of energy consumption.

Clementine L. Chambon et al. [85] applied the sequential antisolvent precipitation technology to extract lignin by proton ionic liquid. Lignin was precipitated by sequentially adding different equivalents of water to the mixture of lignin, which had a more uniform molecular weight distribution than lignin precipitated by directly adding excess water. Lignin fractionation technology can effectively screen the polydisperse phenolic products produced in the biorefinery process and obtain more homogeneous lignin components through fractionation, which shows great potential in the value-added production of lignin industry [86]. Lignin sequential fraction by organic solvents is an emerging technology in recent years, but it is often accompanied by volatile, toxic, and other issues. As a green and efficient solvent system, ionic liquids have shown unique advantages in lignin fractionation. Its adjustable polarity and hydrogen bond alkalinity can selectively dissolve lignin fragments with different molecular weights and chemical structures, providing a new idea for the precise separation and subsequent functional application of lignin.

PILs have lower viscosity and vapor pressure compared to aprotic ionic liquids, are easier to recover, and have a simpler preparation process, which has aroused the research interest of researchers. In the extraction process, the mechanism of action of protic ionic liquids is simpler—inducing β-O-4′ bond cleavage by the proton— and the resulting lignin molecules have lower molecular weight and are more uniform. In addition, PILs hardly dissolve cellulose and can extract lignin selectively. Currently, PILs with different cationic groups have been developed for the extraction of lignin in lignocellulose. However, acidic environments also make lignin C-C condensation. By introducing organic solvents, lignin condensation can be inhibited, which can meet the needs of lignin priority strategies. In addition, the sequential antisolvent precipitation technology of lignin after treatment with ionic liquids also provide new ideas for the value-added utilization of lignin.

### 3.5. Combination Process

Except single ILs pretreatment, in recent years, there have been studies on the combination of two or three pretreatment methods for commercial-scale process development. ILs pretreatment methods can be combined with physical and chemical pretreatment methods [87,88,89,90,91,92,93,94]. Compared with the single ILs pretreatment method, the synergic effect between IL and other methods (e.g., microwave, ultrasonic) will reduce reaction time and enhance the extraction efficiency of lignocellulose.

Combining ILs pretreatment with microwave or ultrasound methods has been studied more in recent years [89,91,92,93]. The mechanism of microwave promoting the fractionation process of proton ionic liquid is attributed to the synergistic effect of ionic liquid and microwave on the depolymerization of lignocellulose through ion conduction. Through dielectric polarization on chemical bonds, microwaves will cause molecular collisions and at last induce the breakdown of lignocellulose. IL can effectively absorb microwave energy and shorten the heating time in a way that is beneficial to energy. Wang et al. [89] established a microwave-assisted [TEA][HSO_4_] ionic liquid fractionation process for corn straw fractionation to promote monosaccharide production and support downstream acetone butanol ethanol (ABE) fermentation. Microwave irradiation can significantly shorten the fractionation cycle of corn straw. Under the optimized conditions (190 W, 3 min), high xylan removal rate (93.17 ± 0.63%) and delignification rate (72.90 ± 0.81%) were achieved. According to Sun et al. [91], compared with the traditional oil-bath heating method (140 °C, 180 min), microwave method (140 °C, 40 min) has a higher lignin yield (45.8%) and shorter reaction time.

In addition, supported ionic liquid membranes have demonstrated significant potential for the selective separation and purification of lignocellulosic components. Abejón et al. [94] investigated the selective permeation of various industrial lignins (including kraft lignin and lignosulfonates) and monosaccharides (xylose and glucose) through different SILM configurations in aqueous environments. The research evaluated five distinct membrane supports and nine ionic liquid combinations, revealing that only the system of 1-butyl-3-methylimidazolium dibutyl phosphate ([Bmim][DBP]) and polytetrafluoroethylene (PTFE) showed selective solute transport ability.

## 4. The Mechanism of Lignin Extraction by Ionic Liquid

A large number of ionic liquids (ILs) have been developed for the extraction of lignin from lignocellulosic biomass. Understanding the mechanisms behind IL-mediated lignin extraction will facilitate the development of more efficient lignin extraction systems. In this section, we summarize current research on the mechanisms of lignin extraction using ILs. The interaction mechanisms between ILs and lignin primarily involve lignin dissolution and degradation.

### 4.1. Dissolution of Lignin in Ionic Liquids

The dissolution of lignin in ILs can be attributed to the hydrogen bonding between the anions of the ionic liquid and lignin, and the hydrogen bond basicity of the anions remains the key factor determining the solubility of lignin in ionic liquids. Unlike cellulose dissolution, the hydrogen bonding basicity of IL anions used for delignification is smaller than that required for cellulose dissolution [55]. In addition, the cations in ILs capable of dissolving lignin showed the ability to interact with the system of aromatic units in lignin, further promoting the dissolution of lignin in ionic liquids.

Except experimental research, many calculation methods (MD, DFT, etc.) have been used to explore the interaction between lignin and ionic liquids. [4,95,96,97,98]. The research results generally support the crucial role of anions in lignin dissolution. However, the cation interaction relationship is still controversial. Jon Zubeltzu et al. [4] used DFT, MD, and AIMD computational simulations to explore the role of cations in the solvation process of lignin in ionic liquids and did not observe the interaction between cations and lignin through π stacking. The calculation results showed that there was a dispersion effect between imidazole side chain and lignin ring and reported for the first time that imidazole-based cations could stabilize lignin hydroxyl and lignin ring. Bharat Manna et al. [96] also supported this view, showing that the imidazole ring interacts with hydroxyl and methoxy groups, and the ethyl tail interacts with the benzene ring of lignin.

In addition, the role of cosolvent in ionic liquids has also been simulated, and the interaction energy relationship between anions, cations, and solvents has been analyzed [96,99,100]. Ge et al. [99] found that a small amount of water (10–20 wt%) would destroy the ion association in IL, reduce the interaction between cation and anion, and help cation and anion be free to participate in lignin dissolution. The excessive addition of water will play an antisolvent role, which is not conducive to the dissolution of lignin. Bharat Manna et al. [100] found that 80% water could not destroy the dense structure of lignin itself, and water hindered the formation of hydrogen bonds between anions and lignin.

### 4.2. The Depolymerization Mechanism of Lignin in Ionic Liquids

The process of IL pretreatment is also accompanied by the depolymerization, dehydration, and recondensation of lignin [101]. After ionic liquid pretreatment, the regenerated lignin was tested by GPC, HSQC, ^13^P NMR, etc., a large number of results showed that ionic liquid pretreatment caused the bond broken between lignin units, the molecular weight of lignin decreased. And lignin showed more moderate dispersion and molecular weight, which was conducive to further value-added utilization. However, its exact mechanism of action is very complex and unclear. In recent years, many studies have proposed ideas on the degradation mechanism of lignin under different conditions [73,101,102,103,104]. The β-O-4′ bond is the most abundant bond in lignin, and its bond dissociation energy is weaker than that of the C-C bond and other C-O bonds. Therefore, the cleavage of the β-O-4′ bond can be regarded as a key step in the depolymerization of lignin [105].

**Figure 5:**
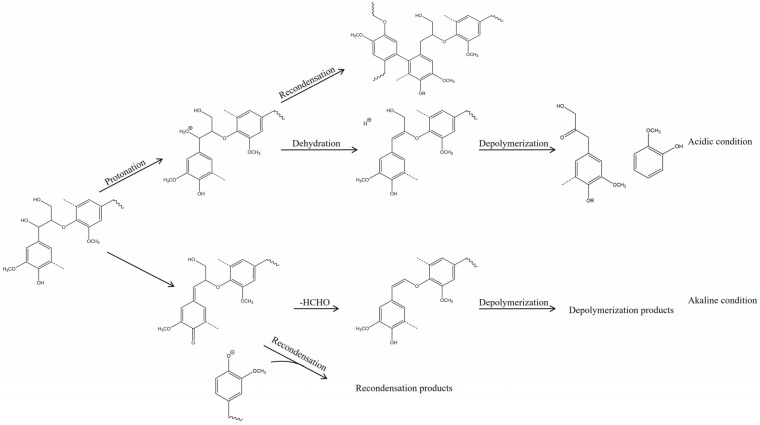
Possible mechanisms of lignin degradation under acidic and alkaline conditions [101].

The pH value of ILs is a critical factor influencing the degradation pathways of lignin. ILs with different acid–base properties exhibit distinct effects. The figure illustrates the acid–base characteristics of several ILs used for lignin dissolution. According to Tanmoy Dutta et al. [101], compared to near-neutral ILs ([C_2_C_1_Im][OAc]) and alkaline ILs ([Ch][Lys]), acidic ILs ([TEA][HSO_4_]) demonstrate a stronger ability to cleave β-O-4′ bonds. In both acidic ([TEA][HSO_4_]) and alkaline ([Ch][Lys]) environments, lignin undergoes significant depolymerization followed by recondensation, whereas in near-neutral ILs ([Bmim][OAc]), only depolymerization occurs without significant recondensation. Acidic and alkaline ILs catalyze lignin depolymerization through different pathways, and Figure 5 presents possible degradation mechanisms under acidic and alkaline conditions. (1) Acidic ionic liquids induce the protonation of hydroxyl groups on α-carbon, initiate the dehydration reaction, form carbocation, and then hydrolyze to cause the breaking of β-O-4′ bond, and finally they generate phenols and carbonyl compounds [73]. This can also explain that the addition of a small amount of water can promote the increase in lignin extraction rate in the acidic ionic liquid pretreatment process [102]. However, the carbon cations generated during the degradation process may also couple with electron-rich sites on aromatic rings, leading to condensation reactions that form β-β bonds and consequently increase molecular weight [106]. (2) The alkaline ionic liquid may induce the β-O-4′ bond, forming a quinone methylate intermediate (VIII), to break, followed by the demethoxylation and dehydration reaction (α-β unsaturated compound), and finally realize lignin degradation. Under an alkaline environment, lignin structural units may undergo a recondensation reaction by forming β-β′ and β-5′ bonds [101]. For near-neutral ionic liquids, the heating method may influence their degradation mechanisms. According to Liu et al. [107], imidazolium-based acetate ILs such as [Emim][Ac] and [Bmim][Ac] under microwave irradiation tend to follow a degradation mechanism more similar to that of alkaline ILs. Sun et al. [91] reported that [C_4_C_1_im]Cl under microwave conditions and [C_2_C_1_im][OAc] under oil-bath heating exhibit degradation mechanisms resembling those of acidic ILs. In contrast, [C_2_C_1_im][OAc] under microwave irradiation behaves similarly to alkaline ILs in lignin degradation.

In addition to the pH value of the ILs causing differences in lignin degradation pathways, the temperature and biomass source during extraction can also affect lignin degradation. Wen et al. [103] explored the depolymerization process of lignin in 1-ethyl-3-methylimidazolium acetate ([C_2_mim][OAc]). [C_2_mim][OAc] selectively degraded the G unit fragment of lignin. However, different lignocellulose showed different degradation trends, and the S unit of Eucalyptus was preferentially degraded over the G unit. The different degradation mechanisms caused by the complexity of biomass unit structure need more targeted research. Temperature also affects the degradation sequence of lignin units. Under high temperature conditions (160 °C), the S unit of eucalyptus trees is easier to remove, while under low temperature conditions (120 °C), the G unit is easier to remove [91].

These studies have provided insights into the mechanisms of lignin depolymerization under different pH values, temperatures, and biomass sources. Future work should integrate advanced characterization techniques such as NMR, FT-IR, and DFT simulations to conduct comprehensive investigations on the depolymerization mechanisms of lignin from various sources under different conditions. Such research will facilitate the broader application of ionic liquids in lignin extraction and valorization.

## 5. Computational Simulation Method for Screening Ionic Liquids

### 5.1. Screening ILs by Computational Simulation Method

The methods commonly used for computational simulations of lignin mainly include quantum chemical (QC) methods, molecular dynamics (MD) simulations, and COSMO-RS [108]. The QC method is a widely used approach for studying molecular structures. It can predict chemical reaction intermediates, reaction barriers, vibrational frequencies, and other thermodynamic parameters by analyzing the trajectories of electron interactions. It primarily employs density functional theory (DFT) and Hartree–Fock (HF) theory [109]. Among these, DFT is more extensively applied, and various solvation models have been developed for DFT to predict intermolecular cooperative effects. However, the QC method is limited to a scale of 1–10 nm [110]. To address this, MD simulations were developed to study atomic trajectories. Force fields serve as the foundation of MD simulations, and currently, all-atom (AA), united-atom (UA), and coarse-grained (CG) force fields have been developed for MD applications [109]. Due to their predictive capabilities at the microscopic level (atoms and electrons), QC methods and MD simulations are often employed to investigate the interaction mechanisms between lignin and ILs. We have already introduced the application of QC and MD in the mechanism of action between lignin and IL in Section 5.

COSMO-RS is a thermodynamic model that calculates the chemical potential of each component in a solution by processing the screening charge density on molecular surfaces. It can effectively address the computational complexity arising from the diverse organic anions and cations in ILs. Recognized as a powerful tool for rapidly screening ILs capable of dissolving lignin, COSMO-RS significantly reduces the time and cost associated with experimental research [111,112,113]. Yu et al. [111] predicted the solubility of 19 common lignin unit models in 3886 ILs through COSMO-RS. Combined with the activity coefficient analysis and solubility experimental verification results, they provided an effective strategy for the appropriate IL screening in the process of lignin depolymerization. Viscosity plays an important role in the dissolution process of lignin. Higher viscosity is not conducive to the mass transfer of lignin and then harms the dissolution of lignin. Moodmohan et al. [112] used the COSMO-RS model to calculate the interaction energy of ionic liquid cation and anion, so as to predict its viscosity change. The results showed that the viscosity of amine, pyridine and phosphine-based ionic liquids was lower than that of choline and imidazole-based ionic liquids, while the increase in alkyl chain length of cations or anions was accompanied by the increase in ionic liquid viscosity.

Current research on ionic liquid screening focuses on the effect of ionic liquid on lignin model. In lignocellulose, lignin often forms complex covalent structure with hemicellulose, and the interaction between lignin and carbohydrate will also affect the lignin extraction rate. Based on this, Karol Baran [113] and others built a machine learning (ML) model to predict the lignin extraction rate in the process of extracting lignin from lignocellulose, which laid the foundation for further designing the process of selective extraction of lignin using ionic liquids.

### 5.2. Computational Simulation Method in Lignin Valorization

Lignin depolymerization is a crucial step in the valorization of lignin, and ILs have been widely applied in this process. In addition to screening ILs capable of dissolving lignin, MD simulations and DFT can enhance the understanding of the depolymerization mechanism of lignin in ILs, thereby facilitating better application of ILs in lignin valorization. Sun et al. [114] employed MD simulations to study the interaction energies in the oxidative depolymerization of lignin catalyzed by copper-containing imidazolium-based ILs. They constructed a structural model of the catalyst–IL–lignin system and calculated the binding energies (*E*_BE_) between [C_4_C_1_im]Cl, CuCl_2_, [C_4_C_1_im]CuCl_3_, and lignin. The *E*_BE_ values reflected the strength of binding interactions at catalytic sites, providing a microscopic explanation for the effectiveness of CuCl_2_ in the oxidative depolymerization of lignin. Zhao et al. [115] conducted DFT simulations on certain PILs and used electrostatic potential (ESP) analysis to predict the active sites for lignin interactions. By examining the ESP distribution maps of various PILs, we could visually assess the electrostatic potential values, thereby predicting the electrostatic interactions with lignin. Benjamin G. Janesko [116] performed DFT calculations on three hexafluorophosphate-based ILs models to investigate the mechanism of acid-catalyzed hydrolysis of a β-O-4 lignin model compound.

### 5.3. Challenges

It can be seen from the above that computational simulation has played an important role in revealing the micromechanism of interaction between ionic liquids, cosolvents and lignin, optimizing and screening solvents, designing new ionic liquids, etc., but there are still limitations and challenges. There are many reasons: on the one hand, because of the complexity of the combination of anions and anions of ionic liquids, on the other hand, because of the complexity of lignin structure (structural heterogeneity, branching and crosslinking, polydispersity, functional group diversity, source variability, and lignin cell wall interaction, etc.) [117]. And at the same time, the synergistic effects of ionic liquids, water and other solvents, as well as lignin and other components of lignocellulose should also be taken into consideration. Various factors may cause the lignin model to be underrepresented, such as the model and natural differences in structure, the model not considering all substances involved in the reaction, etc. In future work, it is necessary to comprehensively consider the above factors to develop a simulation model that is more in line with the actual situation. The simulation results need to be confirmed with experiments (NMR, FTIR, etc.).

## 6. The Application of Ionic Liquids in the Value-Added Utilization of Lignin

**Figure 6:**
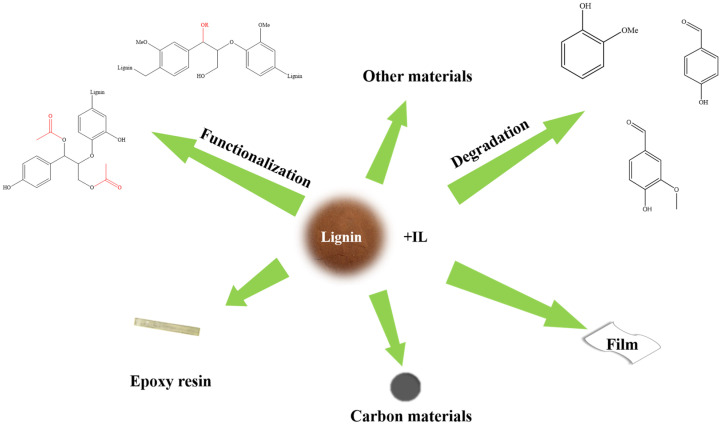
The application of ionic liquids in the value-added utilization of lignin.

The abundant functional groups (hydroxyl, carbonyl, ether bond, etc.) and complex network structure of lignin provide a variety of possibilities for its application. Various anions of ionic liquids endow them with catalytic properties, which can be used as catalysts for ring opening, dehydration, and other reactions. Ionic liquids have also been developed for lignin value-added utilization, such as the preparation of vanillin, epoxy resin, biofuels, membranes, hydrogels, etc. (Figure 6).

### 6.1. Lignin Degradation

Lignin degradation process is generally realized by the bond breaking between lignin units. Ionic liquids of anions, such as Cl^−^, HSO_4_^−^, Ac^−^, MnCl_4_^−^, etc., attack the inter-unit bonds such as β-O-4 and C-C during the reaction, causing the inter-unit bonds to break, and are degraded into small molecule compounds such as syringaldehyde, guaiacol, and vanillin [114,115,118,119,120,121,122,123,124,125,126,127]. HSO_4_^−^ can catalyze the specific production of vanillin from lignin, which is more conducive to the downstream processing and utilization of lignin [118]. Peng et al. [119] showed high efficiency in the continuous flow depolymerization process of lignin using the synergistic effect of N-allylpyridinium hydroxide ([Apy][OH]) and ethylene glycol. Under alkaline conditions, the β-O-4 bond of lignin forms a quinone methyl intermediate, which subsequently breaks to form aromatic monomers. Ethylene glycol can stabilize aldehyde intermediates through an etherification reaction to prevent repolymerization. Ionic liquids act not only as catalysts but also as reaction media in the catalytic depolymerization process, promoting the dissolution and uniform dispersion of lignin [121].

Among various depolymerization methods, oxidative depolymerization is favored by researchers because of its mild reaction conditions and its conversion into high value-added chemicals. Researchers also tried to introduce O_2_ into ionic liquid to catalyze lignin depolymerization reaction and achieved a good catalytic effect. The addition of O_2_ can promote depolymerization and inhibit C-C condensation during depolymerization [122,123,124,125]. POM (polyoxometalate) and 1-butylimidazolium hydrogensulfate ([HC_4_im][HSO_4_]) combine to conduct oxidative depolymerization and inhibit the further oxidation of aldehydes to carboxylic acids by regulating the amount of POM, realizing the directional and efficient conversion of lignin [122]. Kang et al. [123] constructed a binary system of 1-propenyl-3-methylimidazolium bis[(trifluoromethyl)sulfonyl] imide ([Pmim][NTf_2_]) and 1-propylsulfonic-3-methylimidazolium trifluoromethanesulfonate ([PrSO_3_Hmim][OTf]), which can induce the light reaction of lignin to generate free radicals and realize the efficient degradation of lignin. Under UV light irradiation, [Pmim][NTf_2_] promotes Cβ-H bond cleavage through its strong electronegativity, resulting in R·. R· reacting with oxygen to produce peroxy radicals (·OOR). Peroxy radicals further react with water to generate hydroperoxides and hydroxyl radicals. Protons provided by [PrSO_3_Hmim][OTf] promote the decomposition of hydroperoxides, leading to the breaking of C-O-C bonds, and finally the formation of aromatic acids and phenolic products.

Metal-based ionic liquids exhibit a catalytic effect on the depolymerization of lignin [114,126,127]. Kuldeep Singh et al. [126] used 1-butyl-3-methylimidiazolium tetrachloroferrate ([Bmim][FeCl_3_]) to selectively depolymerize the G unit of lignin into vanillin in a room-temperature water medium [FeCl_3_], as a Lewis base can accept lone pair electrons of phenolic hydroxyl oxygen during depolymerization, promoting the breaking of the C-C bond and β-O-4 bond. Sun et al. [114] used [C_4_C_1_im][CuCl_3_] to significantly improve the efficiency of oxidative depolymerization of alkali lignin and selectively convert the H unit of lignin to hydroxybenzaldehyde. During the reaction, [C_4_C_1_im][CuCl_3_] also acts as an electron acceptor to promote the breaking of the ester bond of the H unit.

### 6.2. Lignin Modification

(1) Lignin functionalization

Amino functionalization, epoxide functionalization, carboxylic acid functionalization, ester functionalization, multiple bond functionalization, and hydroxyl functionalization are typical applications of lignin modification [128]. The application of ionic liquids in lignin functionalization has also been reported [129,130,131,132,133,134,135]. Shiori Suzuki et al. [129] reported the process of selective modification of aliphatic hydroxyl groups of lignin. They used [Emim][OAc] as catalyst and solvent to acetylate lignin R-OH and Ar-OH, and deacetylated Ar-OAc groups by taking advantage of the difference in stability of R-OAc and Ar-OAc acetyl groups, thus achieving selective modification of R-OH. Wang et al. [130] used [TEA][OTf] to achieve selective benzyl alkoxylation of lignin at room temperature. Beata Kurc et al. [131] used [C_4_C_1_mim][MeSO_3_] and [C_4_mim][HSO_4_] to selectively oxidize lignin hydroxyl groups to form carbonyl groups. Thaynara C. Pin et al. [132] used PIL synthesized by amine to treat lignocellulose, promoted the amination of lignin, obtained targeted lignin in the pretreatment process, and provided a new way of lignin value-added utilization.

Demethylation modification of lignin is an effective strategy to overcome the obstacles of its high-value transformation. Liu et al. [136] used a novel proton-based ionic liquid (PIL) 1,2-propanediamine/glycolic acid (PD/GA) as a catalyst and solvent to realize the targeted oxidation of lignin. The treated lignin has the greatest UV resistance and antioxidant activity. Luo et al. [137] reported a low-cost proton ionic liquid demethylation method for preparing lignin-based polyphenol materials. These results provide a new way to develop green and efficient demethylation methods of lignin.

(2) Lignin derived materials

ILs treatment of lignin has been widely used to produce various derived materials, such as lignin derived carbon materials (CFS) [138,139,140], composite hydrogel materials [141,142,143], UV shielding materials [54,127], green supercapacitor electrode materials [144], photocured room temperature phosphorescent (RTP) materials [145], high temperature proton exchange membranes in fuel cells [146], epoxy resin composites [78,147], etc. The abundant phenolic hydroxyl groups of lignin endow the functional materials with antioxidant properties, while the complex structure gives the composites excellent mechanical properties. The addition of ionic liquid provides conductivity, and the tunability of structure makes it possible to customize the function of composites.

Samson o. Anuchi et al. [138] reported the co-pyrolysis of [C_2_mim][NTf_2_] and [C_4_mim][OTf] to prepare lignin-derived carbon materials with large surface area, which contain abundant surface groups. Wang et al. [54] prepared the composite membrane with pretreated IL lignin solution and chitosan. The complex structural units and groups of lignin enhanced the mechanical strength, thermal stability, and hydrophobicity of the composite membrane, providing it with antioxidant, antibacterial, and UV resistance functions. Zhang et al. [141] reported a lignin/ionic liquid hydrogel dressing. Lignin has antioxidant activity due to its abundant phenolic hydroxyl groups, while ionic liquids have antibacterial activity and self-healing properties. The combination of ionic liquids and lignin has broadened the application scope of lignin-derived functional materials.

Lignin, as a renewable energy, also has a wide range of value-added applications. Rashid et al. [148] studied the effect of PIL treatment on the pyrolysis products of lignin extracted from oil palm biomass. They found that lignin extracted using the aprotic ionic liquid pyridinium formate (PyFor) yielded higher phenolic and aromatic compound contents with the lowest acid concentration, making it a promising candidate for biofuel production.

Interestingly, lignin-derived ionic liquids show good application potential. Sharon Monaci et al. [149] prepared a series of lignin-based ionic liquids based on choline and found that they showed good application potential in metal corrosion prevention. Yu et al. [150] prepared a lignin-based ionic liquid, which presented excellent lubrication performance. The phenolic hydroxyl groups in lignin can trap oxygen free radicals to provide the material with excellent corrosion resistance. This provides a new idea for the value-added utilization of lignin.

## 7. Conclusions and Future Directions

Lignocellulose is the largest bio-renewable energy storage resource in nature, and its efficient utilization helps to solve the current energy shortage problem facing the world. At present, the research of lignocellulose separation mostly focuses on the extraction and enzymatic hydrolysis of cellulose, and lignin is often produced as an additional product. Effective production and utilization of lignin is beneficial to increase the economic value of lignocellulose.

(1) Ionic liquids have shown great potential in the separation of lignin from lignocellulose. The selective extraction of lignin can be achieved by adjusting the structure of ionic liquid. The lignin-first strategy has become a key focus in the field of biorefining. Current studies place greater emphasis on preserving lignin’s native structure while utilizing cellulose. Adjusting IL structures or incorporating auxiliary agents may help achieve this goal. Future studies should focus on developing IL-based systems in this direction to enable the efficient utilization of lignocellulosic biomass.
(2) Investigating the lignin extraction mechanism by ILs helps us understand IL–lignin interactions and develop more efficient lignin extraction systems. However, the structural complexity and diversity of lignin lead to variations in its degradation mechanisms, and the changes in lignin structure and molecular weight during IL-mediated dissolution still lack detailed and systematic studies. Computational simulations have enhanced our understanding of the mechanisms underlying lignin dissolution and extraction, enabling faster and more effective screening of ILs capable of dissolving lignin. Current computational studies often employ simple lignin models, neglecting the structural complexity of lignocellulose and its impact on simulations. Additionally, few studies have explored simulations of IL extraction systems that preserve lignin’s structural integrity. Future research should refine computational models and expand the application scope of simulation tools.
(3) Compared with organic solvents, the industrial application of ILs is hindered by their higher viscosity and elevated costs. After pretreatment, IL recovery is necessary to reduce cost. Current IL recycling typically involves adding antisolvents to separate ILs from lignocellulose components, but this process incurs non-negligible energy consumption for distillation. Additionally, the relatively high viscosity of ILs limits pretreatment efficiency. In future research, computational simulations could be leveraged to guide the development of IL systems with lower cost, reduced toxicity, and improved viscosity, thereby enhancing their industrial feasibility.
(4) Meanwhile, current recycling processes have problems such as inefficient lignin recovery and low lignin purity. In future studies, we should adopt the lignin-first strategy to optimize ILs pretreatment procedures. Lignin fraction should be implemented to maximize its utilization from lignocellulose, thereby enhancing the overall economic value of lignocellulose.
(5) In addition, ionic liquids also show good application value in lignin value-added utilization. The structural tunability of ionic liquids can meet the specific needs of lignin value-added. However, most of the applications are still in the laboratory and fail to carry out large-scale industrial production. In the future, we should further expand its application scope and promote the transformation of lignin from “industrial waste” to “renewable resources” to achieve greater results.

## Figures and Tables

**Table 1:** Different methods of lignin extraction [29].

Major Pretreatment Methods	Conventional Type	Characteristic
Milling treatment	Physical pretreatment	Reduced the particle size and disrupted the structure of the biomass matrix High energy demand Inefficient when used alone
Microwave treatment	Physical pretreatment	Synergistic with other methods can improve lignin removal efficiency High energy consumption
Acid treatment	Chemical pretreatment	Short reaction period and low process cost Significant removal rate of lignin by breaking glycosidic and aryl ether bonds Might cause lignin condensation Corrosion on the tankage and environment
Alkakine treatment	Chemical pretreatment	Chance to obtain lignin is more in modified form Effective in substantial lignin removal Less recovery of biomass
Organic treatment	Chemical pretreatment	Feasibility of obtaining pure lignin Obtain high purity lignin by co-solvent enhanced lignocellulosic fractionation lignin High volatility with potential explosion hazard
Ionic liquid treatment	Chemical pretreatment	Effective in lignin removal Non-volatility, low melting points, low vapor pressure so biomass is easily treated at high temperature Weak corrosiveness and environmentally friendly Structural adjustability lead different structure of extracted lignin
Hot water treatment	Physicochemical pretreatment	No extraneous addition of chemicals Solid recovery is high Less removal of lignin Significant consumption of energy
Steam explosion treatment	Physicochemical pretreatment	High removal of lignin High temperature and high pressure conditions High process cost
Fungi treatment (Brown Fungi, White Fungi)	Biological pretreatment	Mild reaction condition Lengthy processing time

**Table 2:** PILs used for lignin extraction.

Abbreviation	Name	Structure	Reference	Lignin Yield
[TMG][HSO_4_]	1,1,3,3-tetramethylguanidinium hydrogen sulfate	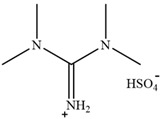	[75]	81%
[DMBA][HSO_4_]	N,N,N-dimethyl butylammonium hydrogen sulfate	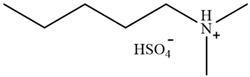	[76]	73%
[TEA][HSO_4_]	Triethylammonium hydrogen sulfate	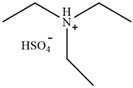	[70]	80%
[DIPEA][Ac]	N,N-Diisopropylethylammonium acetate	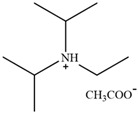	[77]	71.2%
[DIPEA][P]	N,N-Diisopropylethylammonium propanoate	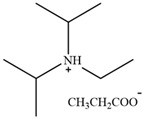	[77]	63.7%
[DIPEA][O]	N,N-Diisopropylethylammonium octanoate	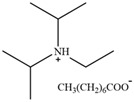	[77]	54.2%
[C_3_H_6_SO_3_Hmim][HSO_4_]	1-(3-sulfopropyl)-3-methylimidazolium bisulfate	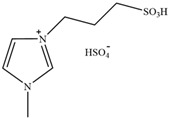	[78]	79.94%
[C_3_SO_3_HMIM][Cl]	1-methyl-3-(3-sulfopropyl)-imidazolium chloride	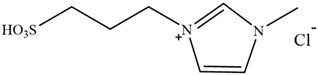	[79]	78%
[Py][Ac]	Pyridinium acetate	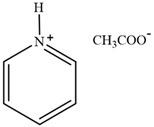	[80]	76%
[Py][For]	Pyridinium formate	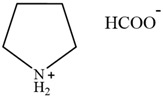	[21]	Not reported
[MEA][Ac]	2-hydroxyethylammonium acetate	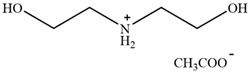	[81]	Not reported
HAc-[EOA][OAc]	Acetic acid-Ethanolamine acetate	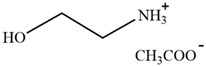	[82]	46%
[N11H(2OH)][LAC]	2-hydroxyethyl ammonium lactate	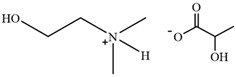	[83]	56%
